# Patient and Operational Factors Do Not Substantively Affect the Annual Departmental Quality of Anesthesiologists’ Clinical Supervision and Nurse Anesthetists’ Work Habits

**DOI:** 10.7759/cureus.55346

**Published:** 2024-03-01

**Authors:** Franklin Dexter, Bradley J Hindman, Emine O Bayman, Rashmi N Mueller

**Affiliations:** 1 Anesthesia, University of Iowa, Iowa City, USA; 2 Biostatistics/Anesthesia, University of Iowa, Iowa City, USA

**Keywords:** work habits, teamwork, supervision, safety climate, performance monitoring, quality control charts, non-technical skills, mixed effects model, confounders, anesthesia

## Abstract

Introduction: Although safety climate, teamwork, and other non-technical skills in operating rooms probably influence clinical outcomes, direct associations have not been shown, at least partially due to sample size considerations. We report data from a retrospective cohort of anesthesia evaluations that can simplify the design of prospective observational studies in this area. Associations between non-technical skills in anesthesia, specifically anesthesiologists’ quality of clinical supervision and nurse anesthetists’ work habits, and patient and operational factors were examined.

Methods: Eight fiscal years of evaluations and surgical cases from one hospital were included. Clinical supervision by anesthesiologists was evaluated daily using a nine-item scale. Work habits of nurse anesthetists were evaluated daily using a six-item scale. The dependent variables for both groups of staff were binary, whether all items were given the maximum score or not. Associations were tested with patient and operational variables for the entire day.

Results: There were 40,718 evaluations of faculty anesthesiologists by trainees, 53,772 evaluations of nurse anesthetists by anesthesiologists, and 296,449 cases that raters and ratees started together. Cohen’s d values were small (≤0.10) for all independent variables, suggesting a lack of any clinically meaningful association between patient and operational factors and evaluations given the maximum scores. For supervision quality, the day’s count of orthopedic cases was a significant predictor of scores (P = 0.0011). However, the resulting absolute marginal change in the percentage of supervision scores equal to the maximum was only 0.8% (99% confidence interval: 0.2% to 1.4%), i.e., too small to be of clinical or managerial importance. Neurosurgical cases may have been a significant predictor of work habits (P = 0.0054). However, the resulting marginal change in the percentage of work habits scores equal to the maximum, an increase of 0.8% (99% confidence interval: 0.1% to 1.6%), which was again too small to be important.

Conclusions: When evaluating the effect of assigning anesthesiologists and nurse anesthetists with different clinical performance quality on clinical outcomes, supervision quality and work habits scores may be included as independent variables without concern that their effects are confounded by association with the patient or case characteristics. Clinical supervision and work habits are measures of non-technical skills. Hence, these findings suggest that non-technical performance can be judged by observing the typical small sample size of cases. Then, associations can be tested with administrative data for a far greater number of patients because there is unlikely to be a confounding association between patient and case characteristics and the clinicians’ non-technical performance.

## Introduction

Although the perioperative safety climate and non-technical skills of operating room staff, including teamwork, likely influence the clinical outcomes of patients after surgery, a systematic review with meta-analysis “failed to find a statistically significant improvement of patient outcomes” with staff training in non-technical skills [1]. This finding was tentative because it was based on “a small number of heterogeneous studies” [1]. In the current article, we use data from a retrospective cohort of anesthesia evaluations to investigate the validity of a simplified design of prospective observational studies of safety climate, teamwork, and perioperative clinical outcomes.

Teamwork in operating rooms can be measured by retrospective self-assessment using several tools [2]. This approach can generate large sample sizes for study. However, the tools have lacked concurrent validity or reliability [2]. In contrast, teamwork in operating rooms can be quantified with validity and precision using trained observers [2]. Such studies are expensive because of the personnel and time needed to perform observations. However, suitably powered clinical trials using trained observers and the Non-Technical Skills Scale assess differences in teamwork with only ≈25 cases in each group (e.g., orthopedic surgery in the pediatric surgical suite) [3,4]. The principal limitation of designing and budgeting these studies to evaluate patient outcomes is the practical impossibility of prospective observation of >1000 cases for measuring patient outcomes [5-7].

Consider a different strategy. For each surgical specialty, observation would be performed for the typical and suitable sample size of two or three dozen cases per group (e.g., orthopedics at one surgical suite) [3,4]. Non-technical skills would be measured, albeit with substantive standard error. Also, the researchers would estimate outcomes of hundreds or thousands of patients using administrative data [5-7]. There, too, will be substantive standard errors because the patients will have heterogeneous outcomes and risks of morbidity. The association between the independent and dependent variables would be estimated using methods incorporating uncertainty in both variables [8-10]. The strategy would produce valid inferences if the measurement error in the independent variable (teamwork) is unrelated to covariates of the dependent variable, specifically patient-related factors. For example, if teamwork consistently was assessed to be higher for sicker patients than healthier patients, this strategy would be invalid. However, a lack of association of teamwork with patient-related conditions is expected because non-technical skills are routinely compared among and between different specialty teams without controlling for other patient-related factors [11]. The conundrum is that establishing a lack of association between teamwork and patient-related factors requires large sample sizes, comprising thousands of patients.

In the current article, we report an unexpected opportunity to test for associations between teamwork and patient-related factors. Tens of thousands of observations have already been performed of two different measures of non-technical operating room clinical performance in anesthesia, to which we then added patient-related and operational data. We use the term “anesthesiologist” to denote physicians who have completed residency and, frequently, fellowship training. The anesthesiologists’ quality of clinical supervision (i.e., their intraoperative role) was evaluated daily using the de Oliveira Filho supervision scale [12-15]. We use the phrase “nurse anesthetist” to denote certified registered nurse anesthetists. Their clinical performance was evaluated daily using a work habits scale [16,17]. Both scales are integrally related to teamwork and safety climate [18,19] and, hence, to the quality of clinical care provided by these practitioners. Evaluation of the overall (pooled) clinical quality of the hospital department’s practitioners can be made validly by combining daily evaluations of the individual practitioners [20]. Changes in an anesthesia department’s overall quality of clinical care, measured annually, have been used to assess the evaluation program, the department’s leadership, and resulting managerial decisions [20]. These changes in quality reflect, in part, the anesthesia practitioners who were newly hired and those who left the hospital (i.e., the ratees being evaluated and the raters performing the evaluations). However, changes in clinical quality may also represent changes in patient-related factors over time (e.g., changes in daily practitioners’ caseloads of very sick patients, such as those with the American Society of Anesthesiologists’ physical status 4 or higher).

The studied anesthesia department has obtained daily evaluations for several years [21-27]. With tens of thousands of rater-ratee days, associations can be examined between the evaluation scores and characteristics of the cases and the patients. The absence of meaningful association would suggest that cohort studies can validly evaluate associations between team activity (measured with error) and the clinical outcomes of patients (also measured with error). In this paper, we show that, indeed, the hypothesis holds that the clinical performance of anesthesia practitioners had no substantive associations with patient or operational factors.

## Materials and methods

The University of Iowa Institutional Review Board determined on December 21, 2023, that this project (#202312373) does not meet the regulatory definition of human subjects research.

The dates studied were United States (US) government fiscal years, October 1, 2015, through September 30, 2016, …, October 1, 2022, through September 30, 2023. These were eight fiscal years, not academic years or calendar years, for two reasons. First, US federal databases, including International Classification of Diseases Version 10 Clinical Modification diagnosis codes, Procedure Coding System codes, and Diagnosis Related Groups change based on the US federal fiscal year. Second, the US fiscal year intervals differ from those used at the studied department for faculty promotions, annual performance reviews, and Ongoing Professional Practice Evaluations. Therefore, as emphasized in the Institutional Review Board project submission, the use of fiscal years increased the protection of the confidentiality of the raters [21].

Trainees’ evaluations of clinical supervision provided by anesthesiologists

Clinical supervision provided by anesthesiologists was evaluated using the de Oliveira Filho scale (Table 1) [12]. We use the term supervision to include all clinical oversight functions directed toward assuring the quality of clinical care whenever the anesthesiologist is not the sole anesthesia care provider (Table 1) [22]. These scores have been used for semi-annual Ongoing Professional Practice Evaluations, annual faculty performance reviews, promotion reviews, and monitoring for abrupt changes in performance [15,21,25,26]. Trainees, principally anesthesia residents and fellows, were requested by email to evaluate the quality of supervision of anesthesiologists with whom they had worked during the preceding day using the de Oliveira Filho supervision scale [12,13]. Each of the nine items was scored: one for “never,” two for “rarely,” three for “frequently,” and four for “always” [12-14,25].

**Table 1:** Supervision scale completed daily by residents and fellows The statements asked are quotations (i.e., used precisely the listed wording). All items were presented in the same sequence. The wording differs from that developed [12] only to the extent that (a) the word “faculty” was used instead of “instructor” and (b) the tense of the verbs was changed to past tense because each evaluation was for a specific date working together [24]. These items used have been reported previously in the methods section of multiple studies [14,19,20,22-25].

Item	Statement asked
1	The faculty provided me timely, informal, nonthreatening comments on my performance and showed me ways to improve
2	The faculty was promptly available to help me solve problems with patients and procedures
3	The faculty used real clinical scenarios to stimulate my clinical reasoning, critical thinking, and theoretical learning
4	The faculty demonstrated theoretical knowledge, proficiency at procedures, ethical behavior, and interest/compassion/respect for patients
5	The faculty was present during the critical moments of the anesthetic procedure (e.g., anesthesia induction, critical events, complications)
6	The faculty discussed with me the perianesthesia management of patients prior to starting an anesthetic procedure and accepted my suggestions, when appropriate
7	The faculty taught and demanded the implementation of safety measures during the perioperative period (e.g., anesthesia machine checkout, universal precautions, prevention of medication errors, etc.)
8	The faculty treated me respectfully, and strived to create and maintain a pleasant environment during my clinical activities
9	The faculty gave me opportunities to perform procedures and encouraged my professional autonomy

Anesthesiologists’ evaluations of nurse anesthetists’ clinical work habits

The anesthesiologists were requested by email to evaluate the work habits of the nurse anesthetists with whom they had worked during the preceding day (Table 2) [16,28]. Each of the six items was scored on a five-point scale [16].

**Table 2:** Work habits scale completed daily by departmental anesthesiologists The items are quoted (i.e., used precisely the listed wording). To create the items from Dannefer et al. [28], item (1), “for sessions,” was changed to “for case(s).” Item (2), “overlooks,” was changed to “overlooked.” Item (3), “unable to explain clearly,” was changed to “did not communicate clearly.” Item (4), “lacks initiative,” was changed to “lacked initiative.” Item (5), “only assumes responsibility,” was changed to “only assumed responsibility.” Item (6), “learning agenda,” was changed to “care.” The six items are not Likert scaled, because respondents are not agreeing or disagreeing on a scale with symmetric anchors (e.g., the anchors are not analogous to strongly disagree versus strongly agree). These items used have been reported previously in the methods of multiple studies [10,16,17,27].

Item	Lowest performance = 1	Radio button choices	Highest performance = 5
1	Consistently seemed unprepared for case(s)	1, 2, 3, 4, 5	Consistently well prepared for cases(s)
2	Overlooked important data and failed to identify or solve problems correctly	1, 2, 3, 4, 5	Identified and solved problems using intelligent interpretation of data
3	Did not communicate clearly his or her reasoning process with regard to solving problem(s)	1, 2, 3, 4, 5	Clearly communicated his or her reasoning process with regard to solving problem(s)
4	Lacked initiative or leadership qualities	1, 2, 3, 4, 5	Took initiative and provided leadership
5	Only assumed responsibility when forced to, and failed to follow through consistently	1, 2, 3, 4, 5	Consistently identified tasks and completed them efficiently and thoroughly
6	Dependent upon others for direction with regard to his or her care	1, 2, 3, 4, 5	Thought and worked independently

Processes for supervision and work habits evaluation

Both clinical supervision and work habit evaluations were made for interactions occurring during patient care throughout the workday, not for individual cases [14,16,25,26]. The rater-ratee pair had to work together for at least one hour for an electronic request to be sent to the rater [14,16,25,26]. These were for interactions during days or nights, either workdays or weekends [14,16,25,26]. These were operating room and non-operating room time-based anesthetics [14,16,25,26]. Clicking on the request hyperlink brought up the scale, preceded at the top of the web page with the picture and name of the anesthesiologist or nurse anesthetist to be evaluated to the left [16,17]. Next were listed the date together with the ratee being evaluated, the surgical suite where cases were performed together, and the primary surgical procedure of each case [16,17]. Then, the nine supervision scale items or the six work habits items were listed. All items in evaluations had to be scored for an evaluation to be submitted. Once an evaluation was submitted, it could not be recalled or changed. Evaluation requests that were not completed in 14 days expired automatically. Completion of an evaluation usually took less than one minute (89%) and reliably less than two minutes (96%) [29]. As an analogy, these evaluations can be thought of as two pilots, each evaluating each other, using valid and reliable scales the day after working together. Evaluations were confidential, partly preserved by providing ratees analyzed summaries only every six months [21].

Anesthesia assignment data

Throughout the studied period, the department used Epic Anesthesia (Verona, Wisconsin) for its information system. Information for each case was the date of service, the American Society of Anesthesiologists’ base units, primary surgical Current Procedural Terminology code used for anesthesia billing, the American Society of Anesthesiologists’ physical status, patient age in years, start time, end time, and surgical suite (Table 3). Because evaluations were requested inconsistently for surgical obstetric cases, including cesarean deliveries, neither evaluations nor cases for the obstetrical surgical suite were included. In the case of non-operating room locations with continuous presence, an anesthesia practitioner (e.g., nurse anesthetist) was included (e.g., cardiac electrophysiology laboratory). The 1.5% of cases with missing physical status were treated as missing with intention, as it is a required field.

**Table 3:** Independent variables contributing to evaluations by trainees of anesthesiologists’ clinical supervision Anesthesiology residents completed the 40,718 evaluations for 90% (36,521), base year residents (“interns”) for 5% (1,959), fellows for 3% (1,287), and non-anesthesia resident rotators for 2% (951). As listed in the header of the first column, entries are listed as mean (standard deviation) and median. For example, in the second row and second column, it is “1.389 (1.761), 1.0.” Among the 40,718 evaluations, the trainee rater and anesthesiologist ratee together performed a mean of 1.389 cases per day performed entirely during daytime hours, with a median of one case. In the fourth column are Cohen’s d standardized differences, comparing each row [30,31]. The Cohen’s d values ≤0.10 are very small [30]. In the sixth through 17th rows of the table are Clinical Classifications Software categories, listed sequentially, obtained using the case’s primary surgical Current Procedural Terminology code [32]. The categories were used to represent the surgical specialties in a generalizable manner. The last four rows in this table are counts of cases for individual surgical suites at the studied hospital. Because these are physical locations at one hospital, they were not generalizable, unlike the preceding rows. Using the variance inflation factor, explained in the Statistical Methods, we checked and confirmed that individual suites had a multivariable linear association with counts of procedure and age (i.e., they were not adding incremental information). The pediatric surgical suite’s variance inflation factor was 3.48. The non-operating room sites’ variance inflation factor was 12.12. When these last four rows were excluded, the maximum variance inflation factor was less than three, the largest being for the counts of cases for 15 to 30 base units (variance inflation factor of 2.76) and cardiac surgery (variance inflation factor of 2.59).

Independent variables, reported as mean (standard deviation) and median, and each referring to the cases of the rating trainee and the ratee anesthesiologist during the evaluated day	One or more of the nine supervision scale items given a response less than four (“always”)	All nine supervision scale items given a response of four (“always”)	Cohen’s d standardized difference
Cases performed entirely during daytime hours, defined as ending between 6:46 AM and 7:14 PM	1.389 (1.761), 1.0	1.308 (1.658), 1.0	0.048
Cases performed at least in part during nighttime hours	0.139 (0.362), 0.0	0.140 (0.369), 0.0	0.004
Cases with the American Society of Anesthesiologists’ base units, 3 to 7, with 3 being the minimum among all cases	1.130 (1.777), 1.0	1.051 (1.661), 0.0	0.047
Cases with the American Society of Anesthesiologists’ base units, 8 to 14	0.286 (0.588), 0.0	0.262 (0.570), 0.0	0.042
Cases with the American Society of Anesthesiologists’ base units, 15 to 30, with 30 being the maximum among all cases	0.112 (0.385), 0.0	0.136 (0.412), 0.0	0.057
Cases with Clinical Classifications Software categories 001 to 009, Neurosurgery	0.112 (0.399), 0.0	0.113 (0.417), 0.0	0.003
Cases with Clinical Classifications Software categories 013 to 021, Ophthalmology	0.041 (0.350), 0.0	0.051 (0.404), 0.0	0.023
Cases with Clinical Classifications Software categories 022 to 035, Otolaryngology	0.136 (0.646), 0.0	0.124 (0.646), 0.0	0.019
Cases with Clinical Classifications Software categories 036 to 042, General thoracic surgery	0.053 (0.286), 0.0	0.052 (0.275), 0.0	0.003
Cases with Clinical Classifications Software categories 043 to 050, Cardiac surgery and transcatheter cardiac procedures	0.067 (0.308), 0.0	0.086 (0.338), 0.0	0.056
Cases with Clinical Classifications Software categories 051 to 063, Vascular and other cardiology procedures	0.095 (0.388), 0.0	0.090 (0.376), 0.0	0.013
Cases with Clinical Classifications Software categories 066 to 099, General surgery	0.181 (0.503), 0.0	0.162 (0.478), 0.0	0.040
Cases with Clinical Classifications Software categories 100 to 118, Urology	0.132 (0.571), 0.0	0.129 (0.577), 0.0	0.005
Cases with Clinical Classifications Software categories 119 to 141, Gynecology	0.095 (0.437), 0.0	0.073 (0.385), 0.0	0.057
Cases with Clinical Classifications Software categories 142 to 164, Orthopedics	0.334 (0.802), 0.0	0.344 (0.824), 0.0	0.012
Cases with Clinical Classifications Software category 218, Psychiatry	0.122 (1.280), 0.0	0.078 (1.049), 0.0	0.040
Cases with all other Clinical Classifications Software categories	0.280 (1.345), 0.0	0.224 (1.131), 0.0	0.048
Hours per case with rater and ratee together	3.537 (2.845), 2.78	3.467 (2.809), 2.65	0.025
Cases with the American Society of Anesthesiologists physical status 1 or 2	0.836 (1.323), 0.0	0.794 (1.313), 0.0	0.032
Cases with the American Society of Anesthesiologists physical status 3	0.534 (0.885), 0.0	0.477 (0.813), 0.0	0.069
Cases with the American Society of Anesthesiologists physical status 4 to 6	0.137 (0.392), 0.0	0.158 (0.416), 0.0	0.051
Cases with the American Society of Anesthesiologists physical status missing, treated as informatively, because absent for urgent cases	0.021 (0.146), 0.0	0.020 (0.145), 0.0	0.007
Cases with patients aged 0 to 2 years	0.107 (0.487), 0.0	0.123 (0.504), 0.0	0.032
Cases with patients aged 3 to 17 years	0.163 (0.571), 0.0	0.184 (0.605), 0.0	0.036
Cases with patients aged 18 to 64 years	0.834 (1.294), 0.0	0.757 (1.210), 0.0	0.063
Cases with patients aged 65 to 79 years	0.352 (0.665), 0.0	0.315 (0.625), 0.0	0.058
Cases with patients aged 80 years or older	0.072 (0.278), 0.0	0.069 (0.278), 0.0	0.012
Count of ratees with whom rater worked that day (i.e., number of distinct anesthesiologists who supervised the trainee rater)	1.392 (0.620), 1.0	1.390 (0.616), 1.0	0.004
Cases performed in adult inpatient (“main”) surgical suite	0.987 (1.089), 1.0	0.878 (1.043), 1.0	0.104
Cases performed in adult ambulatory surgery center	0.156 (0.757), 0.0	0.218 (0.896), 0.0	0.071
Cases performed in the pediatric surgical suite, including adjacent pediatric cardiac catheterization suite	0.205 (0.813), 0.0	0.209 (0.809), 0.0	0.005
Cases performed at non-operating room sites, including urology cystoscopy suite	0.181 (1.348), 0.0	0.143 (1.138), 0.0	0.032

Our study sample size was the number of evaluations (Table 3). The sample size was not the number of cases, because evaluations were for the entire day, not the case. We list Table 3 here so that readers can refer to specific independent variables and their associated counts of evaluations. The rater and the ratee usually worked together for more than one case during the day (Table 3), an occasion together that resulted in one evaluation for that day. A criterion was needed for assigning cases to evaluations because there were more cases than evaluations (Table 3). Cases were matched to evaluations using the rater and ratee assigned and caring for the patient at the start of the case, defined as within the first 14 minutes. That criterion for matching was used because our long-term goal after the current project was to understand how changes in anesthesia staff assignment may affect patient outcomes. Most assignment decisions were made before cases started (i.e., the decision being who will care for which patients). Using only the anesthesia practitioners signed in at the precise start of the anesthetic was not sufficient for supervising anesthesiologists’ sign-in because the start means the beginning of continuous anesthesia presence, not the induction of anesthesia. Anesthesiologists were included, whether they were medically supervising (trainees or student nurse anesthetists) or “immediately available” for cases of nurse anesthetists. Therefore, the independent variables were counts of cases started together (Table 3). For example, suppose that one day an anesthesiologist and a resident started three cases together, two general surgery cases followed by a vascular surgery case. Then, referring to the eighth through 12th rows of Table 3, one record would have two listed for general surgery, one for vascular surgery, and zeros for otolaryngology, general thoracic surgery, and cardiac surgery, respectively. Among the 296,449 cases, 60 cases (i.e., 0.020%) contributed to the time of a trainee-anesthesiologist evaluation and to the time of the anesthesiologist-nurse anesthetist interaction. That would occur either if the nurse anesthetist helped the resident and anesthesiologist start the case or, as was more common in the department workflow, a senior anesthesia resident carrying the emergency pager helped the anesthesiologist and nurse anesthetist start the case. Needing to assign each of these 60 cases to one or the other, each was assigned to the rater who had the fewest evaluations during the studied year.

Statistical analyses

Data analyses were performed using Stata version 18.0 (StataCorp, College Station, Texas). Standardized differences were presented in terms of Cohen’s d [30,31]. They were calculated using the Stata command “stddiff” [31]. The variance inflation factor is a measure of collinearity among variables. The variance inflation factor was calculated using the Stata “estat vif” command. When one or more of the variables have a variance inflation factor greater than 10, it shows the presence of large multicollinearity. These calculations of variance inflation factor were performed after one variable had been excluded from each category of variables because otherwise there was complete collinearity. For example, every case had either 3 to 7, 8 to 15, or 16 to 30 American Society of Anesthesiologists’ base units (Table 3). Consequently, the variance inflation factor was calculated using the count of cases with 8 to 15 units and using the count of cases with 16 to 30 units, but not the count of cases with three to seven units, because that would be redundant as the total count of cases was the sample size of cases.

**Figure 1:**
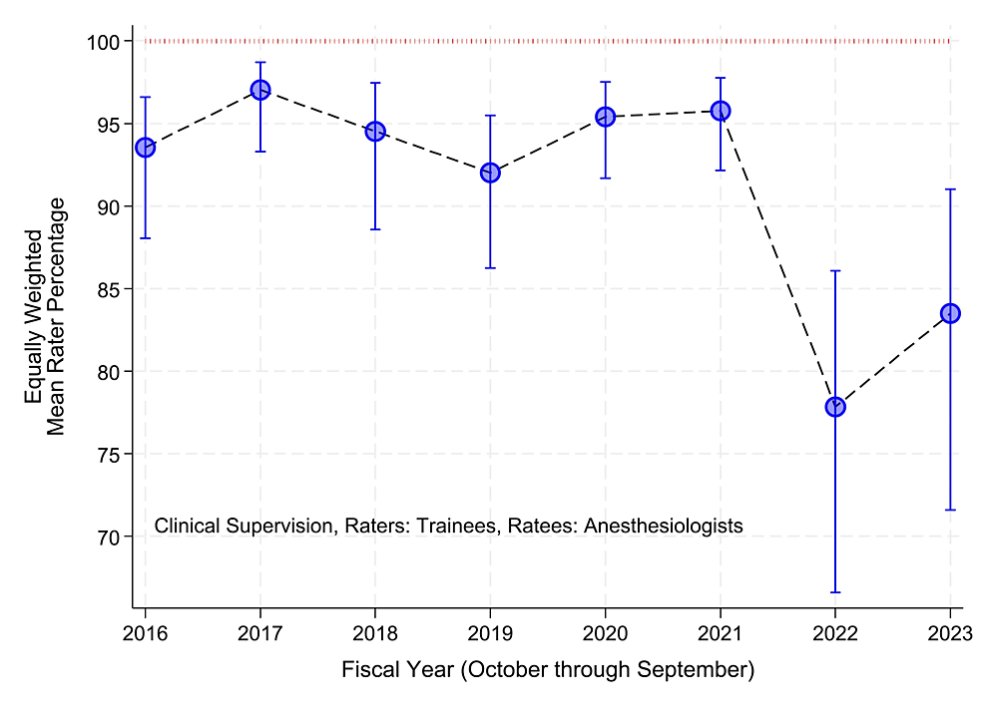
Variation among years in the overall annual departmental quality of anesthesiologists’ clinical supervision of trainees The mixed effects logistic regression model was applied separately to data from each year. The vertical axis gives the percentages of evaluations with all nine items (Table 1) given the maximum rating. Using the mixed effects logistic regression model with no fixed effects other than the intercept, each year’s pooled estimate was calculated with equal weighting of each rater. The error bars represent 99% two-sided confidence intervals. The times are fiscal years (e.g., “2022” means October 1, 2021, through September 30, 2022). Note that the standard errors in the logit scale were no greater in the last two years than in earlier years (i.e., the confidence intervals are wider because the point estimates for those two years were closer to 50%).

**Figure 2:**
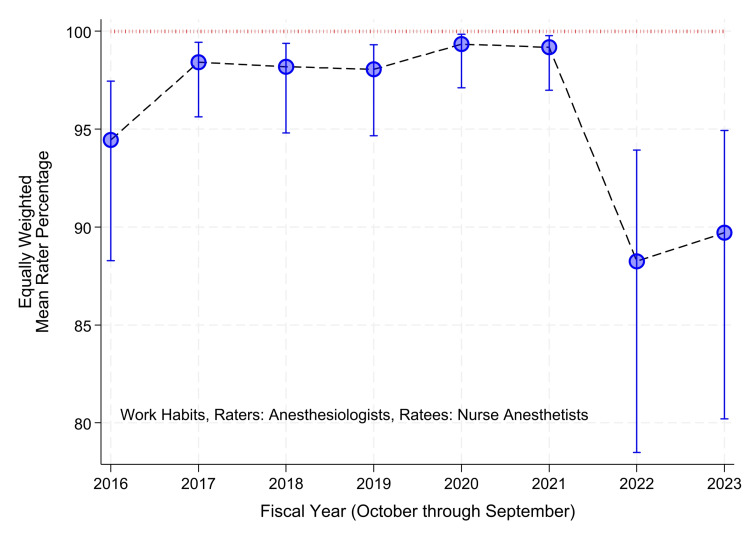
Variation among years in overall annual departmental quality of nurse anesthetists’ work habits The mixed effects logistic regression model was applied separately to data from each year. The vertical axis gives the percentages of evaluations with all six items (Table 2) given the maximum rating. Using the mixed effects logistic regression model with no fixed effects other than the intercept, each year’s pooled estimate was calculated with equal weighting of each rater. The error bars represent 99% two-sided confidence intervals. The times are fiscal years (e.g., “2023” means October 1, 2022, through September 30, 2023). We discuss the similar pattern among years in scores between Figure 1 and Figure 2 later in the Limitations section. Note that the standard errors in the logit scale were smaller in the last two years than in earlier years. In other words, heterogeneity in raters’ scores was reliably no greater. The confidence intervals were wider because the point estimates for those two years were closer to 50%.

The figures were produced using the steps developed and tested previously, using mixed effects logistic regression model with the only fixed effect being the intercept, and random effects being the raters [20]. A binary variable was assigned, equaling one if all items in the supervision scale, or work habits scale, were equal to the maximum and zero otherwise [17,20,25]. Thus, one was a favorable score, and zero was less than a favorable score. Earlier studies showed that, in practice, analyzing the data as binary does not cause a loss of information [17,20,25]. For example, the distributions of mean scores among raters had marked negative (left) skewness [20]. The resulting confidence intervals for the skewed means were impracticably wide, and their interpretation suggested a lack of validity [20]. Both limitations were rectified when analyzed in the logit domain [20]. The analyses were performed separately by year, using the Stata "melogit" command [20]. The logits of the binary variable followed a normal distribution among raters [20]. The overall departmental performance for each fiscal year was estimated using the intercept-only mixed effects logistic regression model with random effects of the raters [20]. The random effects model was used so that each rater had an equal weight [20]. The inverse logit was taken of the estimated intercept, for each year, as well as for its 99% lower confidence limit and its 99% upper confidence limit, to transform the estimates back to the probability scale [20]. The choice of 99% confidence intervals, rather than 95%, was made to achieve some adjustment for the multiplicity [20]. The estimates and intervals are plotted in Figure 1. The same mixed effects model was applied to evaluations of nurse anesthetists’ work habits. These are plotted in Figure 2. The mixed effects logistic regression method detects changes in overall department performance over the years [20].

The new analyses of the current paper were to add all the independent variables from Table 3 to the mixed effects regression model, doing so with the random effects being the combinations of years and raters. Thus, differences among years have been adjusted in the single mixed effect model, for each of the two scales, as desired. The fixed effects for each evaluation were the characteristics of the cases performed by the rater-ratee combination that day. P < 0.01 was treated as statistically significant, and 99% confidence intervals were calculated for screening variables for statistical significance. Because there were at least 23 fixed effects, depending on how they were counted, P-values also were reported with adjustment for the false discovery rate using the Benjamini and Hochberg procedure [33]. The effect size of each of the statistically significant variables was estimated using the predictive marginal estimate of the slopes of the probability of all items in the respective scale being the maximum with respect to the variables, centered at their means, and with all other variables at their observed quantities. The Stata “margins dydx” command was used. Two sensitivity analyses were performed to evaluate the reliability of the results. One sensitivity analysis was to estimate the slope centered at the median of the variable. The other sensitivity analysis was to add a fixed effect for each year. Because the random effects included the rater-year combination, these estimates for fixed effects had large standard errors. We did not also use mixed effects logistic regressions with two random effects, raters nested by year, because the effects of years were fully inconsistent with being independent and identically distributed random variates in the logit scale (Figures 1, 2).

## Results

We used 94,440 evaluations, of which 40,718 were evaluations of faculty anesthesiologists by trainees (Table 3), and 53,772 were evaluations of nurse anesthetists by anesthesiologists (Table 4). To obtain the 40,718 evaluations by trainees, 58,665 requests were sent, 27% (16,123) were not completed, and 3% (1,824) reported that there had been too minimal interaction with the ratee for evaluation. To obtain the 53,722 evaluations by nurse anesthetists, 64,288 requests were sent, 15% (9,393) were not completed, and 2% (1,173) reported minimal interaction with the ratee. The evaluations were completed in a mean of 4.0 days (standard deviation of 3.7 days) and a median of 3.0 days. For 85% of the evaluations of clinical supervision, all nine items were given the maximum response (34,780/40,718) (Figure 1). For 83% of the evaluations of work habits, all six items were given the maximum response (44,536/53,722) (Figure 2).

**Table 4:** Independent variables contributing to evaluations by anesthesiologists of nurse anesthetists’ work habits In the fourth column are Cohen’s d standardized differences, comparing each row [30,31]. The Cohen’s d values ≤0.10 are very small [30]. In the sixth through 17th rows of the table are Clinical Classifications Software categories, listed sequentially, obtained using the case’s primary surgical Current Procedural Terminology code [32]. The categories were used to represent the surgical specialties in a generalizable manner. The last four rows are variables for individual surgical suites at the studied hospital. These were not generalizable, unlike the preceding rows. Using the variance inflation factor, explained in the Statistical Methods, we checked and confirmed that individual suites had a multivariable linear association with counts of procedure and age (i.e., they were not adding incremental information). The pediatric surgical suite’s variance inflation factor was 4.82. The non-operating room sites’ variance inflation factor was 14.77. When the last four rows of variables were excluded, the maximum variance inflation factor was less than three, the largest being for the counts of cases for ophthalmology (variance inflation factor of 2.03).

Independent variables, reported as mean (standard deviation) and median, and each referring to the cases of the rating anesthesiologist and the ratee nurse anesthetist during the evaluated day	One or more of the nine supervision scale items given a response less than four (“always”)	All nine supervision scale items given a response of four (“always”)	Cohen’s d standardized difference
Cases performed entirely during daytime hours, defined as ending between 6:46 AM and 7:14 PM	2.032 (1.547), 2.0	2.039 (1.740), 2.0	0.004
Cases performed at least in part during nighttime hours	0.080 (0.276), 0.0	0.084 (0.282), 0.0	0.013
Cases with the American Society of Anesthesiologists’ base units, 3 to 7, with 3 being the minimum among all cases	1.802 (1.632), 1.0	1.837 (1.812), 1.0	0.020
Cases with the American Society of Anesthesiologists’ base units, 8 to 14	0.296 (0.604), 0.0	0.274 (0.576), 0.0	0.037
Cases with the American Society of Anesthesiologists’ base units, 15 to 30, with 30 being the maximum among all cases	0.015 (0.123), 0.0	0.011 (0.113), 0.0	0.027
Cases with Clinical Classifications Software categories 001 to 009, Neurosurgery	0.119 (0.416), 0.0	0.137 (0.453), 0.0	0.040
Cases with Clinical Classifications Software categories 013 to 021, Ophthalmology	0.307 (1.067), 0.0	0.256 (0.979), 0.0	0.051
Cases with Clinical Classifications Software categories 022 to 035, Otolaryngology	0.191 (0.708), 0.0	0.204 (0.739), 0.0	0.017
Cases with Clinical Classifications Software categories 036 to 042, General thoracic surgery	0.023 (0.202), 0.0	0.023 (0.188), 0.0	0.004
Cases with Clinical Classifications Software categories 043 to 050, Cardiac surgery	0.022 (0.172), 0.0	0.020 (0.170), 0.0	0.008
Cases with Clinical Classifications Software categories 051 to 063, Vascular and cardiology	0.122 (0.484), 0.0	0.116 (0.463), 0.0	0.012
Cases with Clinical Classifications Software categories 066 to 099, General surgery	0.200 (0.540), 0.0	0.194 (0.527), 0.0	0.012
Cases with Clinical Classifications Software categories 100 to 118, Urology	0.169 (0.640), 0.0	0.212 (0.762), 0.0	0.058
Cases with Clinical Classifications Software categories 119 to 141, Gynecology	0.116 (0.511), 0.0	0.113 (0.502), 0.0	0.007
Cases with Clinical Classifications Software categories 142 to 164, Orthopedics	0.551 (0.984), 0.0	0.511 (0.937), 0.0	0.042
Cases with Clinical Classifications Software category 218, Psychiatry	0.052 (0.817), 0.0	0.110 (1.224), 0.0	0.050
Cases with all other Clinical Classifications Software categories	0.290 (0.987), 0.0	0.336 (1.324), 0.0	0.036
Hours per case with rater and ratee together	2.771 (1.692), 2.33	2.721 (1.677), 2.27	0.030
Cases with the American Society of Anesthesiologists physical status 1 or 2	1.241 (1.275), 1.0	1.267 (1.376), 1.0	0.020
Cases with the American Society of Anesthesiologists physical status 3	0.730 (0.974), 1.0	0.717 (1.044), 0.0	0.012
Cases with the American Society of Anesthesiologists physical status 4 to 6	0.112 (0.338), 0.0	0.110 (0.342), 0.0	0.004
Cases with the American Society of Anesthesiologists physical status missing, treated as informatively, because absent for urgent cases	0.030 (0.182), 0.0	0.028 (0.172), 0.0	0.011
Cases with patients aged 0 to 2 years	0.128 (0.484), 0.0	0.135 (0.506), 0.0	0.014
Cases with patients aged 3 to 17 years	0.277 (0.689), 0.0	0.294 (0.701), 0.0	0.024
Cases with patients aged 18 to 64 years	1.158 (1.252), 1.0	1.156 (1.394), 1.0	0.001
Cases with patients aged 65 to 79 years	0.448 (0.779), 0.0	0.438 (0.781), 0.0	0.013
Cases with patients aged 80 years or older	0.101 (0.333), 0.0	0.101 (0.338), 0.0	0.003
Count of ratees with whom rater worked that day (i.e., distinct anesthesiologists who worked with a nurse anesthetist)	2.515 (1.323), 2.0	2.424 (1.244), 2.0	0.072
Cases performed in adult inpatient (“main”) surgical suite	0.914 (1.084), 1.0	0.901 (1.067), 1.0	0.013
Cases performed in adult ambulatory surgery center	0.746 (1.498), 0.0	0.640 (1.415), 0.0	0.074
Cases performed in the pediatric surgical suite, including adjacent pediatric cardiac catheterization suite	0.329 (0.909), 0.0	0.350 (0.942), 0.0	0.022
Cases performed at non-operating room sites, including urology cystoscopy suite	0.123 (0.958), 0.0	0.232 (1.386), 0.0	0.083

Some independent variables were statistically significant in the mixed effects logistic regression models (Tables 5, 6). However, Cohen’s d values were very small (≤0.10) for all independent variables, suggesting the lack of clinically meaningful differences by univariate analyses (Tables 3, 4) [30,31]. For evaluations of anesthesiologists’ clinical supervision, the count of orthopedic cases was a statistically significant predictor of scores (Table 5). We estimated the average marginal effect, centered at its mean of 0.34 cases (Table 3). The absolute marginal change in the percentage of supervision scores equal to the maximum was too small to be of clinical or managerial importance (Figures 1, 2). The increase was only 0.8% (99% confidence interval: 0.2% to 1.4%). For evaluations of nurse anesthetists’ work habits, neurosurgical cases may have been a significant predictor of scores (Table 6). We again estimated the average marginal effect, centered at the variable’s mean, 0.13 cases (Table 4). The marginal change was also too small to be of clinical or managerial importance, an increase of only 0.8% (99% confidence interval: 0.1% to 1.6%). As one set of sensitivity analyses, we repeated the calculation of the marginal changes when centered at the variables’ medians of 0 cases (Tables 3, 4). Point estimates and confidence intervals were not different to within 0.1%. As a second set of sensitivity analyses, we repeated the calculations, including the fiscal years as a fixed effect, to ensure no substantive change in estimates. The same two variables were the only ones that were statistically significant. The estimated average marginal changes and confidence intervals were, again, not different, to within the 0.1% digits.

**Table 5:** Mixed effects logistic regression for supervision scores using the independent variables in Table 3 The analysis has 40,718 observations and 484 combinations of rater and year. Odds ratios greater than one mean that the variable was associated with more frequent clinical supervision scores equaling the maximum for all nine items. The variables are listed in the sequence of Table 3. For each category of variables that sum to the total count of cases (e.g., daytime hours in the first row of Table 3 plus nighttime hours in the second row of Table 3), one row is absent because those combined sum to the total. Among the independent variables, one was statistically significant based on the criterion of P <0.01, orthopedic surgery in the 13th row. P = 0.0011 equals P = 0.025 with an adjustment for the false discovery rate.

Independent variable	Odds ratio	99% confidence interval	Unadjusted P-values
Cases performed at least in part during nighttime hours, defined as the case ending before 6:46 AM or after 7:14 PM	0.979	0.863 to 1.110	0.66
Cases with the American Society of Anesthesiologists’ base units, 8 to 14	0.971	0.886 to 1.065	0.42
Cases with the American Society of Anesthesiologists’ base units, 15 to 30	1.068	0.893 to 1.277	0.35
Cases with Clinical Classifications Software categories 001 to 009, Neurosurgery	1.005	0.889 to 1.137	0.92
Cases with Clinical Classifications Software categories 013 to 021, Ophthalmology	1.051	0.921 to 1.201	0.33
Cases with Clinical Classifications Software categories 022 to 035, Otolaryngology	1.000	0.913 to 1.096	0.99
Cases with Clinical Classifications Software categories 036 to 042, General thoracic surgery	0.933	0.772 to 1.127	0.34
Cases with Clinical Classifications Software categories 043 to 050, Cardiac surgery	1.090	0.881 to 1.348	0.30
Cases with Clinical Classifications Software categories 051 to 063, Vascular and cardiology	0.971	0.851 to 1.107	0.56
Cases with Clinical Classifications Software categories 066 to 099, General surgery	1.008	0.913 to 1.114	0.83
Cases with Clinical Classifications Software categories 100 to 118, Urology	0.996	0.909 to 1.091	0.90
Cases with Clinical Classifications Software categories 119 to 141, Gynecology	1.027	0.920 to 1.147	0.53
Cases with Clinical Classifications Software categories 142 to 164, Orthopedics	1.089	1.018 to 1.165	0.0011
Cases with Clinical Classifications Software category 218, Psychiatry	0.986	0.940 to 1.034	0.43
Hours per case with rater and ratee together	0.991	0.975 to 1.008	0.18
Cases with the American Society of Anesthesiologists physical status 3	0.944	0.880 to 1.014	0.039
Cases with the American Society of Anesthesiologists physical status 4 to 6	0.971	0.842 to 1.120	0.59
Cases with the American Society of Anesthesiologists physical status missing, treated as informatively, because absent for urgent cases	1.003	0.742 to 1.356	0.98
Cases with patients aged 0 to 2 years	0.985	0.876 to 1.107	0.73
Cases with patients aged 3 to 17 years	0.927	0.842 to 1.020	0.040
Cases with patients aged 65 to 79 years	0.993	0.910 to 1.083	0.83
Cases with patients aged 80 years or older	1.074	0.906 to 1.274	0.28
Count of ratees with whom rater worked that day (i.e., number of distinct anesthesiologists who supervised the trainee rater)	1.015	0.939 to 1.098	0.61

**Table 6:** Mixed effects logistic regression for work habits scores using the independent variables in Table 4 The analysis has 53,722 observations and 614 combinations of rater and year. Odds ratios greater than one mean that the variable was associated with more frequent work habits scores equaling the maximum for all six items. The variables are listed in the sequence of Table 4. For each category of variables that sum to the total count of cases (e.g., daytime hours in the first row of Table 4 plus nighttime hours in the second row of Table 4), one row is absent because those combined sum to the total. One variable was statistically significant based on the criterion of P <0.01, neurosurgery in the 4th row. P = 0.0054 equals P = 0.12 with an adjustment for the false discovery rate.

Independent variable	Odds ratio	99% confidence interval	Unadjusted P-values
Cases performed at least in part during nighttime hours, defined as the case ending before 6:46 AM or after 7:14 PM	1.021	0.871 to 1.196	0.74
Cases with the American Society of Anesthesiologists’ base units, 8 to 14	0.996	0.913 to 1.087	0.92
Cases with the American Society of Anesthesiologists’ base units, 15 to 30	0.745	0.514 to 1.078	0.040
Cases with Clinical Classifications Software categories 001 to 009, Neurosurgery	1.129	1.009 to 1.264	0.0054
Cases with Clinical Classifications Software categories 013 to 021, Ophthalmology	0.986	0.930 to 1.046	0.55
Cases with Clinical Classifications Software categories 022 to 035, Otolaryngology	0.986	0.914 to 1.064	0.63
Cases with Clinical Classifications Software categories 036 to 042, General thoracic surgery	0.949	0.754 to 1.194	0.56
Cases with Clinical Classifications Software categories 043 to 050, Cardiac surgery and transcatheter cardiac procedures	0.787	0.589 to 1.051	0.033
Cases with Clinical Classifications Software categories 051 to 063, Vascular and other cardiology procedures	1.036	0.931 to 1.152	0.39
Cases with Clinical Classifications Software categories 066 to 099, General surgery	0.994	0.907 to 1.089	0.86
Cases with Clinical Classifications Software categories 100 to 118, Urology	1.014	0.940 to 1.093	0.64
Cases with Clinical Classifications Software categories 119 to 141, Gynecology	1.014	0.931 to 1.104	0.68
Cases with Clinical Classifications Software categories 142 to 164, Orthopedics	0.949	0.897 to 1.004	0.016
Cases with Clinical Classifications Software category 218, Psychiatry	1.030	0.977 to 1.087	0.15
Hours per case with rater and ratee together	0.988	0.958 to 1.018	0.29
Cases with the American Society of Anesthesiologists physical status 3	0.984	0.930 to 1.042	0.47
Cases with the American Society of Anesthesiologists physical status 4 to 6	0.954	0.834 to 1.090	0.36
Cases with the American Society of Anesthesiologists physical status missing, treated as informatively, because absent for urgent cases	0.898	0.707 to 1.141	0.25
Cases with patients aged 0 to 2 years	0.998	0.895 to 1.112	0.96
Cases with patients aged 3 to 17 years	1.058	0.970 to 1.153	0.094
Cases with patients aged 65 to 79 years	1.030	0.958 to 1.108	0.29
Cases with patients aged 80 years or older	0.994	0.867 to 1.138	0.90
Count of ratees with whom rater worked that day (i.e., distinct nurse anesthetists who worked with an anesthesiologist)	0.985	0.946 to 1.026	0.35

## Discussion

Before the current study was performed, we knew that when evaluating anesthesia clinical performance, the year is a key covariate, treated as a categorical variable not continuous, and as a distinct fixed effect for each department [20]. That was expected because departments change over time, including changes in leadership, policies, personnel (i.e., raters and ratees), and the process of evaluation (e.g., instructions). Our results confirm that the fiscal year should be included in statistical models relating anesthesia clinical performance and patient outcome. The type of year used would be the fiscal year because the Diagnosis Related Groups, procedure codes, and diagnosis codes are adjusted (in the United States) by the fiscal year. However, our results also show that other patient and operational variables are unlikely to be important because they had negligible or no significant association with the measures of clinical performance by the anesthesia practitioners involved.

Multiple investigations have studied the effect of anesthesia case assignments, principally breaks and permanent handoffs at the end of cases, on patient outcomes and organizational performance [34-48]. Only one of these 15 studies, which examined turnover times [38], incorporated control for the specific people or a measure of their performance as a mediating variable (e.g., quality of clinical supervision or work habits). Instead, the independent variables were permanent handoff or not, the duration of the workday, counts of cases of the personnel, and so forth. Our group has previously shown that the quantity (counts) of cases was not associated with a higher quality of clinical supervision or work habits [16,23]. Therefore, the lack of consideration of the individual practitioner in most studies shows our current study's importance. From our results, the clinical performance of individual anesthesiologists or nurse anesthetists can be added validly as a single fixed effect. The alternative of entering the practitioners individually as random effects is not a valid substitute because clinicians are assigned non-randomly to cases (e.g., based on preferences) [10,14,29]. Our conclusions are especially likely to be valid because every anesthesiologist working with a trainee knew daily that an evaluation request would be sent, just as every nurse anesthetist knew that the anesthesiologist working with them would be sent an evaluation request (i.e., being rated was not a special occasion to result in behavior change).

As suggested in the Introduction, our results can help guide future studies of operating room teamwork. Investigators in the field of patient safety could have more confidence in our conclusions' applicability to teamwork studies [1,3] if we had 94,440 prospective observations scored using the Non-Technical Skills Scale. Nevertheless, our current data with this large sample size provide indirect insight. One suitable next step would be for already completed clinical trials with prospective observations of non-technical performance as the primary endpoint to be analyzed retrospectively with administrative data from the hospitals for patients from the same time periods and surgical suites. The observational scores are compared with the outcomes of all patients in the studied groups, using statistical methods suitable when there is substantive measurement error in the independent variable (i.e., teamwork, safety climate, etc.) [8-10].

Comparison to other studies

The quality of clinical supervision and anesthesia work habits are unidimensional constructs, associated with non-technical skills [16,49], teamwork [18], and safety climate [18]. However, neither the supervision scale (Table 1) nor the work habits scale (Table 2) is the Non-Technical Skills Scale. They are measuring related but different constructs. Ideally, future research would repeat the current study at different hospitals and with different instruments. Realistically, however, the information from the current study likely will need to suffice for the near future because such data with tens of thousands of observations are very uncommon, and such sample sizes were necessary (Tables 5, 6). On the other hand, our results are consistent with earlier results. Jackson et al. reviewed measurement issues in hospital safety climate surveys [50]. Themes of importance included who performed the evaluations (i.e., analogous to raters being anesthesiologists or trainees) and the hospital type, but not the characteristics of the patients receiving the care [50]. Similarly, Nwosu et al. measured safety climate in operating rooms [51]. Principal factors were who performed the evaluations and the hospital [51]. Gillespie et al. examined the non-technical skills of surgical teams before and after hospital relocation and a substantial increase in workload [4]. During a period of substantial improvement in non-technical skills, all teams achieved comparable improvements in performance [4]. These earlier study results [4,50,51] are consistent with the finding that time and hospital are important but not patient-related factors. The consistency shows the importance of our current article because, unlike these earlier studies [4,50,51], our sample size of tens of thousands was sufficient to test reliably the validity of the assumption.

Earlier studies in the same department evaluated some of the covariates (Tables 3, 4) for the different purposes of whether they needed to be included when comparing among anesthesiologists [14] and among nurse anesthetists [16] with their peers. The dates of the earlier study of the supervision scale did not overlap with the dates of the current study [14]. Anesthesiologist supervision scores provided by residents were negligibly different when the rated anesthesiologist had more American Society of Anesthesiologists’ relative value guide units of work that same day with other residents or nurse anesthetists (Kendall’s tau was −0.057, with a standard error of 0.014) [14]. Multiple types of regression trees to predict supervision scores included no staff assignment variables [14]. The work habits scale’s covariates were assessed, including an overlap of the first three months of the current study’s eight years of data [16]. There were no significant effects of cases started together, percentage of cases with children 12 years or younger, percentage of cases with the American Society of Anesthesiologists’ physical status 4 to 6, percentage of cases with the American Society of Anesthesiologists’ 8 or more relative value guide base units, and percentage of cases performed in the ambulatory surgery center (which at the time of that earlier studied included pediatric patients) [16].

Limitations

We evaluated the association of equally weighted supervision scores and work habit scores on patient factors (Tables 5, 6). That was different from what might be provided if raters had been asked intermittently (e.g., annually) to evaluate the overall department [22]. In an earlier study, all 39 anesthesiology residents gave an overall (global) departmental supervision score, compared with each rater’s average daily score [22]. The overall evaluation score was 14% less than the mean of individual faculty scores, a ratio that was uniformly distributed among raters, and not correlated with multiple potential covariates [22]. Every resident gave an overall departmental evaluation of supervision that was the same or less than the mean of their individual evaluations [22]. The implication is that when evaluating clinical supervision, and probably work habits, pooling daily evaluations to obtain overall departmental performance should not be treated the same as scores obtained by requesting annually that practitioners provide cross-sectionally an overall departmental performance assessment [22].

Although there was a quantitatively very small impact of specialty on supervision and work habits, those variables were examined. The finding of greater work habits for neurosurgical cases was not statistically significant with adjustment for the false discovery rate (Table 6). Therefore, we doubt its reliability. In contrast, the adjusted P = 0.025 for supervision and orthopedic cases (Table 5) suggests that residents perceived closer supervision during regional anesthetic procedures. A limitation is that we can only speculate on the cause.

We do not know the cause of the variation in supervision and work habits scores over the years (e.g., the decrease in clinical quality scores of both anesthesiologists and nurse anesthetists starting in 2022) (Figures 1, 2). Regardless, the change and its cause(s) would have no direct effect on our results because analyses (Tables 5, 6) were performed with rater-year combinations as a random effect. However, without knowing the mechanism of the change, we do not know if there could be an indirect effect both on the independent variables considered and on scores (e.g., via staff assignments). Whether the clinical supervision scores are used for Ongoing Professional Practice Evaluation or annual faculty review, the purpose is to compare performance among clinicians [26]. In an earlier study, we examined mathematically the Shannon information content of the ratings [27]. With a mixed-effects logistic regression model, the raters being fixed effects and the ratees being the random effects, as used semi-annually [17,25], the information content of each rater’s evaluations can be quantified using binomial entropy [27]. The raters with all scores of all items in a year equaling the maximum had no binomial entropy of responses, meaning that the information content of their evaluations was zero [27]. As a result of that study, in the first quarter of the 2022 fiscal year, we started to send email feedback weekly to individual raters when their evaluations were not providing information content [27,52]. That feedback may have changed raters’ scoring behaviors. Thus, it is possible the decrease in scores starting in 2022 was caused by changes in raters’ scoring behaviors rather than ratees’ clinical quality. However, simultaneously, there were multiple leadership changes in the department such that we cannot infer validly with the retrospective data what caused the change.

## Conclusions

Eight fiscal years of departmental evaluation data were used to examine the associations between anesthesiologists’ clinical supervision and nurse anesthetists’ work habits and multiple patient-related factors. Cohen’s d values were very small for all independent variables, suggesting a lack of a clinically meaningful difference by univariate analysis. A few specialty-specific variables were statistically significant. However, the absolute marginal changes in the percentage of supervision scores and work habits scores equal to the maximums were too small to be of clinical or managerial importance. These findings are useful because they show that unadjusted analyses would be suitable when evaluating the association between clinical anesthesia performance and patient outcomes, albeit incorporating uncertainty in both variables. To the extent that our findings apply to non-technical operating room skills in general, not only to anesthesiologists and nurse anesthetists, the results provide a practical study design option to study associations with patient outcomes.
